# Knowledge, attitude, and practices toward COVID-19 among the international travelers in Thailand

**DOI:** 10.1186/s40794-021-00155-1

**Published:** 2021-11-15

**Authors:** Suttiporn Prapaso, Viravarn Luvira, Saranath Lawpoolsri, Archin Songthap, Watcharapong Piyaphanee, Wiwat Chancharoenthana, Sant Muangnoicharoen, Punnee Pitisuttithum, Pornthep Chanthavanich

**Affiliations:** 1 Department of Clinical Tropical Medicine, Faculty of Tropical Medicine, Mahidol University, Bangkok, Thailand; 2 Department of Tropical Hygiene, Faculty of Tropical Medicine, Mahidol University, Bangkok, Thailand; 3 Department of Community Health, Faculty of Public Health, Naresuan University, Phitsanulok, Thailand; 4 Department of Tropical Pediatrics, Faculty of Tropical Medicine, Mahidol University, Bangkok, Thailand

**Keywords:** Knowledge, Attitude, Practices, Coronavirus disease 2019, Travelers

## Abstract

**Background:**

International travel is among the leading impactful factors of COVID-19 transmission; thus, adequate knowledge, good attitude and good preventive practices toward COVID-19 for international travelers are particularly essential for successful pandemic control.

**Methods:**

A cross-sectional, questionnaire-based study was conducted to determine knowledge, attitude and practices (KAP) of international travelers (both Thai and non-Thai) and expatriates in Thailand. The data were collected at the Thai Travel Clinic, Bangkok, Thailand and via online platforms during May to October 2020. The independent T-test, Chi-square test and multiple regression analysis (MRA) were applied to determine factors influencing the KAP.

**Results:**

Of 399 travelers, 46.6% were male, 72.1% had a Bachelor’s degree or higher, and the mean age was 35.6 ± 9.6 years. Due to unexpected travel restrictions and lock down, 77.9% of participants were Thai and the respective major purpose of travel was business/work. Travel cancellation/postponement was reported at 73.9%. While sufficient knowledge (≥ 60% correct answers) was reported in 77.9% of participants, a low percentage of correct answers was found in the questions regarding disease transmission. The travelers reported a neutral attitude and an overall moderate concern regarding the COVID-19 situation. Adequate preventive practices were determined by the average practice score 3.54 ± 0.38 (0 = never and 4 = always). The MRA revealed that the factors influencing good practices were travelers who: i) enrolled from outside the hospital (online platform); ii) received pretravel advice at hospital; iii) were female; iv) participated before the declaration of the end of the outbreak; v) were aged 40–49 years, and vi) visited friends and relatives.

**Conclusions:**

The majority of travelers in this study had sufficient knowledge, a neutral attitude and adequate preventive practices toward COVID-19. The factors influencing good practices included pretravel advice, sex, age and the point in the timeline of the outbreak. In order to better control the COVID-19 pandemic situation, pretravel counselling and advice should be promoted as a means to improve knowledge, particularly in disease transmission, increase awareness and emphasize appropriate preventive measures toward COVID-19 among international travelers. Furthermore, preventive practices should be bolstered at all times regardless of the outbreak situation.

**Supplementary Information:**

The online version contains supplementary material available at 10.1186/s40794-021-00155-1.

## Background

In mid-December 2019, Coronavirus disease 2019 (COVID-19), a new emerging disease from “severe acute respiratory syndrome coronavirus 2 (SARS-CoV-2)”, was identified in Wuhan city, Hubei province, China. Since then, the disease has spread globally and wreaked havoc on all communities in its path, leading to a global pandemic within three months. Despite extensive attempts to control the outbreak, the number of COVID-19 cases increased exponentially in more than 100 countries and has accounted for millions of deaths worldwide.

Before the COVID-19 era, international travel was immensely popular, with a yearly continuous increase in figures. However, international travel is among the most significant contributing factors toward COVID-19 spreading during the pandemics infancy. Consequently, travel-related control protocols, including border closures, travel restrictions, vigorous exit and entry screening and traveler quarantine, have been implemented in tandem with policies to permit only essential travel in several countries as a measure to contain the pandemic. Despite the Thai Department of Disease Control having declared the response protocol against COVID-19 on January 4, 2020, the first confirmed COVID-19 case outside China was reported in Thailand a week later. She was a 61-year-old Chinese woman traveling from Wuhan as an international tourist [1]. A taxi driver who made contact with the sick Chinese traveler, was later diagnosed and confirmed as the first local transmission COVID-19 case [2]. An accelerated increase in cases prompted the Thai government to declare a state of emergency, impose a national lockdown, suspend all international flights and establish a quarantine system for international travelers upon arrival at the end of March 2020 [3].

COVID-19 is an imminent threat toward global health while international travel is a factor that promotes disease transmission. Thus, traveler behavior plays a significant role in disease control and mitigation. Inappropriate awareness regarding COVID-19 may have some ramifications in term of a detrimental pandemic situation while appropriate knowledge regarding COVID-19 may assist with pandemic prevention. Since travel restrictions and quarantine measures may not be practical long-term as COVID-19 solutions, enhancing traveler knowledge and improving attitude which lead to effective preventive practices against the disease should be incorporated. Although studies have been conducted on knowledge, attitude, and practices (KAP) regarding COVID-19 in various populations, including health care workers in China, university students in Japan, and border communities in Northern Thailand [4–17], data for the traveler population have yet to be explored. Hence, this study aimed to investigate the KAP toward COVID-19 among international travelers in Thailand and identify the factors influencing the level of knowledge and preventive behaviors throughout the pandemic. As expatriates are collectively travelers, the KAP of expatriates living in Thailand were also studied.

## Materials and methods

### Study design and population

A cross-sectional, questionnaire-based study was conducted. The study population included international travelers and expatriates in Thailand. All participants were older than 18 years with sufficient understanding of the language within the questionnaire (English or Thai) and were willing to participate in the study. Notably, both Thai and non-Thai participants who had traveled internationally within three months before the interview or planned to travel within one month after answering the questionnaire were recruited.

### Study questionnaire

A bilingual (Thai and English) questionnaire consisted of four parts: i) demographic data and general information related to travel; ii) knowledge; iii) attitude and iv) practices. The knowledge was assessed using 12 multiple-choice questions (four transmission, two signs and symptoms, one diagnostic and five COVID-19 prevention). The attitude part consisted of 16 “5-level Likert scale” questions, eliciting attitude toward COVID-19 transmission, complications, treatments, prevention, and control measures. The participants were also requested to provide a score regarding their concern over the COVID-19 situation, ranging from 0 to 10 (none to maximum concern). The practice part contained ten questions, which inquired as to the frequency of the practices in COVID-19 preventive methods. The reliability of the questionnaire was evaluated among 44 respondents who were not included in the study. Moreover, five experts also verified the questionnaire content validity.

### Data collection

All eligible travelers at the Thai Travel Clinic, the Hospital for Tropical Diseases, Bangkok, were approached, invited, informed and consented to participate in the study. This clinic is the biggest travel clinic in Thailand and is a part of GeoSentinel surveillance sites, which was established in 2005 to provide comprehensive health care for travelers and offer a residency training program in Travel medicine.

In addition to data collection at Thai Travel Clinic, a link to the online questionnaire was also distributed via social media platforms (Facebook and LINE) to recruit eligible participants outside the hospital. This participant pool included limited access to online application groups of expatriates, soldiers commissioned for overseas deployment, and state quarantines as well as the Thai Travel Clinic Facebook page.

### Study timeline

**Fig. 1:**
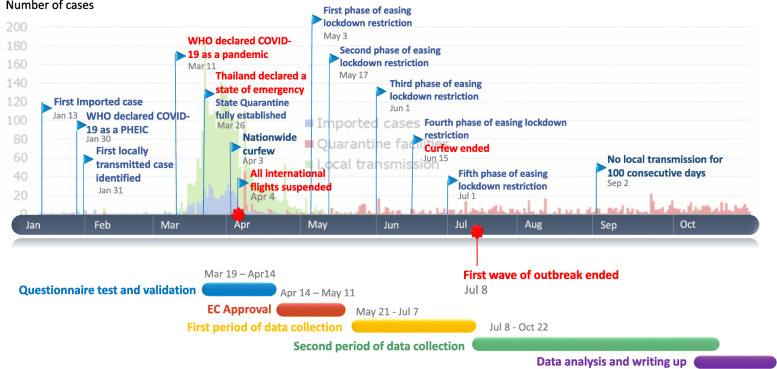
Timeline of the study and Thailand’s COVID-19 situation. The number of COVID-19 cases is plotted in the timeline bar graph and includes Thailand’s COVID-19 situation whereby important events are described. The study timeline is also exhibited below

The timeline of the study and Thailand’s COVID-19 situation during the study period is displayed in Fig. 1. The first wave of the COVID-19 outbreak began in early March 2020 and subsequently reached peak levels by the end of the same month at which point a national lockdown was enforced. This investigative study was conceived in mid-March, had completed the questionnaire testing and validation phase by early April, and recruited participants from May 21, onwards at which point the local community outbreak was controlled and very few COVID-19 cases were prevalent. In August 2020, non-Thai international travelers were allowed to enter Thailand.

Data collection time was divided by the end of the first community COVID-19 outbreak (declared on July 8) into two periods, as seen in Fig. 1. Data collection was completed in October before the second wave of the outbreak began, in December 2020.

### Statistical analysis

With no prior published data associated to this study, a sample size of 385 was calculated based on the assumption that 50% of participants have a sufficient knowledge score regarding COVID-19 with a 95% confidence interval. Data were analyzed using SPSS statistics software version 23. Descriptive statistics, an independent T-test and the Chi-square test were used. The multivariable logistic regression was used to determine the association between potential factors and level of knowledge. A sufficient knowledge was defined as getting the correct answer in ≥60% (≥ 8 from 12 questions). The attitude was assessed using a 5-level Likert scale with answers ranging from 1 (strongly disagree) to 5 (strongly agree). The practices were given a score ranging from 4 (always) to 1 (never). A *p*-value below 0.05 was regarded as statistically significant.

## Results

The total number of participating travelers in the study was 411. Data were then rechecked and validated; 12 travelers were excluded because they had traveled back to Thailand for > 90 days. Therefore, a total of 399 participants were included for data analysis. The site of data collection is displayed in Additional file 1.

### General characteristics of participants and their travels

Of the 399 participants, 224 (56.1%) participants were enrolled during the first period of the study (May–June) and 149 (37.3%) participants were enrolled at the Thai Travel Clinic. The demographic data of the participants are displayed in Table 1. The most common purpose of travel was business/work. Of note, 40% of travelers had experienced the test for COVID-19 and two of them had positive results. In addition, 90% of participants sought pretravel advice, and 54.4% sought a pretravel consultation at hospitals/clinics.

**Table 1:** Demographic data of participants (*N* = 399)

Parameters	N (%)
Date of data collection	
May–June	224 (56.1%)
July - Oct	175 (43.9%)
Site of data collection	
Thai Travel Clinic (inside hospital)	149 (37.3%)
Outside hospital	250 (62.7%)
Age (mean ± SD)	35.6 ± 9.6
18–29	117 (29.3%)
30–39	169 (42.4%)
40–49	76 (19%)
50 or above	37 (9.3%)
Gender: Male	186 (46.6%)
Type of traveler	
Thai	331 (77.9%)
Non-Thai	88 (22.1%)
Occupation	
Healthcare	31 (7.8%)
Non-healthcare	256 (64.1%)
Unemployed	112 (28.1%)
Purpose of travel	
Leisure	81 (20.3%)
Business/work	175 (43.9%)
Visiting friends or relatives	46 (11.5%)
Study	60 (15%)
Other	37 (9.3%)
Education	
Secondary school or lower	111 (27.9%)
Bachelor’s or higher	288 (72.1%)
Influenza vaccination in past 1 year	
Yes	172 (43.1%)
Previous COVID-19 testing	
Yes	162 (40.6%)
Seeking pretravel advice/recommendation	
Yes	361 (90.5%)
Pretravel consultation at hospitals/clinics	
Yes	217 (54.4%)

Major sources of information about COVID-19 were social media (86.5%) and TV/radio (65.7%). Approximately three-quarter of participants reported that the COVID-19 pandemic had greatly affected their travel plans and resulted in trip cancellations or postponements (Table 2). The most common reason accounting for half of the cancellation and/or postponement was the travel restrictions from departure airports or the destination countries.

**Table 2:** Effects of COVID-19 on travel plan

Questions	N (%)
Does COVID-19 affect your travel plan?	
A lot	293 (73.4%)
Some	75 (18.8%)
A little	23 (5.8%)
None	8 (2.0%)
How does COVID-19 affect your trip?	
Trip cancellation	101 (25.3%)
Trip postponement	196 (48.6%)
Minor plan change	64 (16.0%)
No effect	13 (3.3%)
No trip planned	27 (6.8%)
The main reason for trip cancellation or postponement? (*)	
Travel restriction in home country	211 (52.9%)
Travel restriction at destination country	194 (48.6%)
My employer did not allow it	65 (16.3%)
I was worried about the situation	121 (30.3%)
I did not cancel or postpone my trip	33 (8.3%)
I did not have any trip planned	16 (4.0%)
I had to work from home	16 (4.0%)

*multiple answers

### Knowledge on COVID-19

Twelve knowledge questions and the corresponding number of participants with correct answers are shown in Table 3. The knowledge score ranged from 4 to 12, and the mean was 8.64 ± 1.58. The mean knowledge score was significantly higher among travelers whose data were collected outside the hospital (8.79 ± 1.55 vs 8.39 ± 1.60, *p*-value < 0.001), among those who were Thai (8.73 ± 1.55 vs 8.34 ± 1.65, *p*-value 0.041), had a Bachelor’s or higher education level (8.89 ± 1.47 vs 8.02 ± 1.67, *p*-value < 0.001), had been tested for COVID-19 earlier (8.84 ± 1.41 vs 8.51 ± 1.67, *p*-value 0.035), or had a history of influenza vaccination in the past year (8.95 ± 1.45 vs 8.41 ± 1.64, *p*-value < 0.001). The mean knowledge score was also significantly higher in the group whose data were collected in the first period of the study (8.79 ± 1.57 vs 8.46 ± 1.58, *p*-value 0.042) (See Additional file 2).

**Table 3:** Percentages of participants with correct answer

Questions	N (%) – correct answer
1. Which of the following is not a route of Coronavirus spread?	
Mosquito	317 (79.4%)
2. What are the common symptoms of COVID-19?	
Fever, cough, and difficulty breathing	379 (95%)
3. Which of the following is the most reliable way to test for COVID-19 infection during the first week of illness?	
Testing for genetic material from nasopharyngeal swab	354 (88.7%)
4. Which group is at the lowest risk of developing severe disease?	
Children	144 (36.1%)
5. In order to prevent the spread of COVID-19, if you have only one mask, who should get this mask?	
Patient with cough	322 (80.7%)
6. Which one is the most appropriate mask for COVID-19 patient?	
Surgical mask	186 (46.6%)
7. How can you prevent COVID-19 infection?	
Washing hands with alcohol	388 (97.2%)
8. Which of the following is the least useful preventive measure?	
Wearing gloves	339 (85%)
9. After having been in close contact with a confirmed case of COVID-19, how long should you have a self-quarantine if you do not have any symptoms?	
14 days	378 (94.7%)
10. If the red dot is a patient infected with COVID-19, which passenger is at the lowest risk of getting infected?	
A (3 rows from the index case)	85 (21.3%)
11. How long should you wash your hands for to reduce the spreads of coronavirus?	
≥ 20 s	374 (93.7%)
12. Who would be identified as a close contact of COVID-19 patient?	
People who share a dining table with the patient but use serving spoons	183 (45.9%)

Sufficient knowledge regarding COVID-19 (scored ≥60%) was reported in 77.9% of participants. The factors that were associated with sufficient knowledge were further determined using the logistic regression analysis. (Table 4) The covariates with *p*-value < 0.1 in univariate models were included in the multivariable analysis. The factors independently associated with a sufficient knowledge score were type of travelers (Thai) and education level (Bachelor’s degree or above).

**Table 4:** Factors associated with sufficient knowledge

	Knowledge			
	Sufficient* N (%)	Insufficient N (%)	OR (95%CI)	Adjusted OR (95%CI)
Site of data collection				
Thai Travel Clinic	107 (71.8%)	42 (28.2%)	1	1
Non-hospital areas	204 (81.6%)	46 (18.4%)	1.74 (1.08–2.81)	1..21 (0.69–2.10)
Traveler				
Thai	216 (69.5%)	95 (30.5%)	1	1
Non-Thai	15 (51.7%)	14 (48.3%)	0.38 (0.17–0.84)	0.51 (0.21–1.23)
Expatriate	34 (57.6%)	25 (42.4%)	0.48 (0.26–0.89)	0.45 (0.23–0.89)
Education				
Secondary or lower	56 (68.5%)	35 (37.5%)	1	1
Bachelor’s or higher	235 (81.6%)	53 (18.4%)	2.04 (1.24–3.36)	2.16 (1.28–3.65)
Influenza vaccination in the past 1 year				
No	129 (56.8%)	93 (43.2%)	1	1
Yes	136 (79.1%)	36 (20.9%)	1.85 (1.12–3.05)	1.50 (0.88–2.55)
Previous COVID-19 testing				
No	144 (60.8%)	93 (39.2%)	1	1
Yes	121 (74.7%)	41 (25.3%)	1.62 (0.98–2.68)	1.17 (0.66–2.07)

### Attitude toward COVID-19

**Fig. 2:**
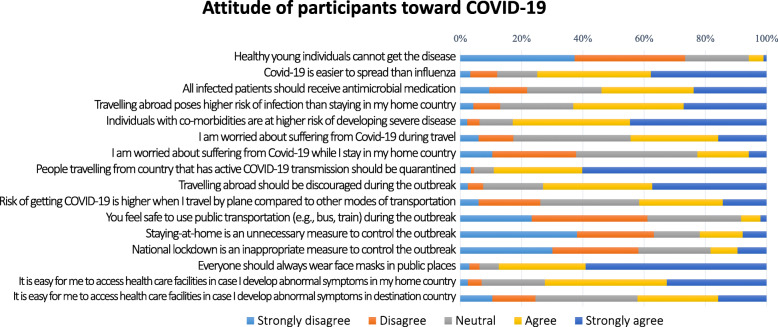
Attitude of the participants toward COVID-19. The attitude of international travelers toward COVID-19 was assessed with “5-level Likert scale” questions. The percentages of the attitude of participants regarding COVID-19 are displayed in the diverging stacked bar chart

The attitude toward COVID-19 is displayed in Fig. 2. Nearly 80% of participants had agreed that COVID-19 was easier to spread than influenza and international travel should be discouraged throughout the outbreak. Approximately 60% of participants agreed that international travel posed greater risk than domestic travel. Over 80% of participants consider it is necessary for travelers from COVID-19 active countries to quarantine and mask-wearing to be mandatory in public. Seventy percent of participants agreed that their respective home country’s healthcare facilities were easily accessible in an event they developed abnormal symptoms. However, only 41% agreed that easily accessible healthcare facilities were available if abnormal symptoms arose at their destination countries.

The mean concern score over the COVID-19 situation was 6.09 ± 2.32 (maximum concern = 10). There were no significant differences in the mean score between the two data collection periods (*p*-value 0.703).

### Practices

**Fig. 3:**
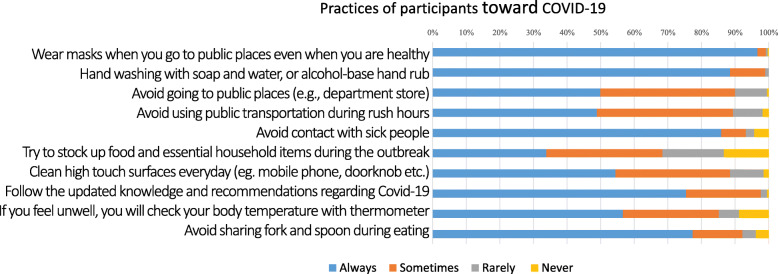
Practices of the participants toward COVID-19. The percentages of participants’ practices of ten preventive measures toward COVID-19 are plotted in the stacked bar graph

Although the overall mean practice score was high, a wide variation in the practice rate of each preventive measure was reported. The practices for reducing COVID-19 transmission were assessed by their frequency within several preventive parameters, ranging from 4 (always) to 1 (never). The mean practice score was 3.54 ± 0.38. The rate of ‘always practices’ by item ranged from 33.8–96.5% (Fig. 3). Almost all participants reported regular practices of basic preventive procedures against COVID-19. The rate of ‘always practices’ was high in the categories of wearing a mask when going to public places (96.5%), hand washing (88.5%) and avoiding contact with sick people (86%). However, only half of the participants reported good practices in the categories of avoiding going to public areas/using public transportation during rush hour, cleaning high touch surfaces every day, and checking body temperature when they feel unwell.

To determine factors influencing practices regarding COVID-19 prevention, the stepwise multiple regression analysis (MRA) was employed. With respect to sociodemographic characteristics, the independent influencing factors for practices in COVID-19 prevention were site of data collection, pretravel advice at hospital, gender, period of data collection, age, and purpose of travel. (Table 5) The results revealed that mean practice score tended to be higher in participants who completed the questionnaire from outside the hospital (Beta = 0.195), participants who sought pretravel advice (compared with those who did not) (Beta = 0.168) and females (Beta = 0.154). In contrast, participants in the second period of data collection tended to have lower mean practice score (Beta = − 0.153). Participants aged 40–49 years had higher mean practice score compared to those who were younger or older (Beta = 0.143). Lastly, participants who visited friends and relatives were more likely to have higher mean practice score than other travel purposes (Beta = 0.107).

**Table 5:** Factors influencing practice on COVID-19 (Stepwise MRA)

Independent variables	b	Beta	*p*-value
Site of data collection (outside hospital)	0.152	0.195	< 0.001
Pretravel advice at hospital (Yes)	0.127	0.168	< 0.001
Gender (female)	0.116	0.154	0.001
Period of data collection (second)	−0.116	−0.153	0.003
Age (40–49 years)	0.138	0.143	0.002
Purpose of travel (VFR)	0.126	0.107	0.022
Constant	2.903		< 0.001

R = 0.415 R^2^ = 0.173 F = 13.621 *p*-value < 0.001

VFR = visiting friends and relatives

The differences in the practices of participant groups were then evaluated by item using the Chi-square test, and the percentage of participants who regularly (always) practice preventive measures were compared with those who do not regularly (sometime/rarely/never) practice (Table 6). Overall, the results showed a higher trend of ‘always practices’ in the first period than the second period of data collection. Most participants always wore a mask when going to public places, and there were no differences between different groups except Thai and non-Thai travelers (98.1% vs 90.9%, *p*-value 0.001). The hand hygiene practice was significantly higher in females and in the first period of data collection. Furthermore, the practice of cleaning high touch surfaces everyday was significantly more common among females, lower education group, Thai travelers, and among participants in the first period of data collection.

**Table 6:**
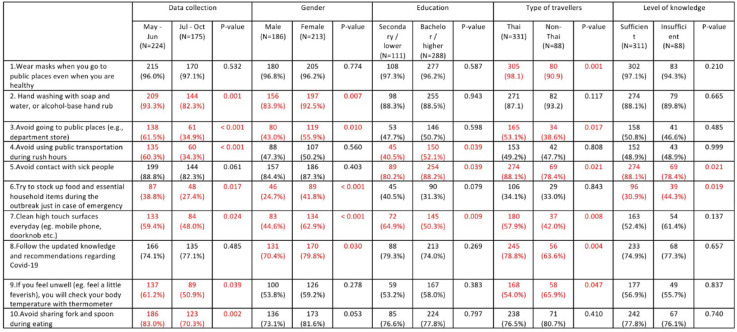
The difference between participant groups and practices of COVID-19 measured by each item (Chi-square test)

## Discussion

With international travel as a major contributing factor toward COVID-19 transmission, appropriate disease preventive behaviors within traveler groups is crucial for pandemic control. For this reason, we investigated traveler knowledge and attitudes toward COVID-19 in addition to the prevalence of preventive protocol practices regarding COVID-19 among travelers and expatriates in Thailand. Although the KAP relating to COVID-19 had been assessed in various populations [4–14, 16–18], our original study pioneers elicited COVID-19 KAP data among the travelers. However, the unexpected lockdown and travel restrictions may have had an effect on study populations. Of note, most of the participants were Thai, aged 18–39, non-healthcare workers, had a high level of education and traveled for essential reasons (only 20% for leisure).

In terms of COVID-19 knowledge, the study revealed a high percentage of participants with good knowledge which was similar to those reported in several studies [4–8]. However, our findings were different from a previous KAP study in the border population of Northern Thailand at the very beginning of the outbreak which found that 73% of participants had poor knowledge [9]. The difference in results could be explained by both the baseline characteristics of participants and the period of the study. The factors that were associated with a sufficient knowledge score were: i) a higher education level (Bachelor’s or above) which was consistent other studies [6, 9, 11–14] and ii) the nationality, possibly due to too few non-Thai travelers in our study. Comparable with the other KAP studies [4, 8, 10, 11], social media was the major source of knowledge concerning COVID-19. Therefore, improving reliability and maintaining up-to-date COVID-19 information on social media would contribute to better traveler knowledge. Interestingly, participants had relatively lower percentages of correct answers in questions pertinent to transmission when compared to symptoms and signs/diagnosis and prevention. This result indicates an opportunity to improve education toward COVID-19 transmission.

In terms of attitude, most participants had a neutral attitude toward COVID-19. The level of concern over the COVID-19 situation was also neutral (average score 6.09 ± 2.32 out of 10) perhaps due to the point in the timeline of COVID-19 at which the observation took place. Indeed, the study was conducted when the COVID-19 situation in Thailand was under control with no local transmission identified, and all the control measures in Thailand had been eased. Interestingly, even though our study participants were international travelers, most of them agreed that traveling abroad should be discouraged during the outbreak. The reason for this finding might be that the COVID-19 situation during the time of data collection was not very active in most countries, and thus some participants tended to be more aware of imported cases.

Regarding prevention practices, the study reported an adequate mean practice score and this score was higher in the group where data were collected during the first period (before the end of the outbreak was declared in Thailand). This finding might be explained by the variation risk perception at that point in time. We hypothesized that when the outbreak was declared to be over, people might reduce their preventive behaviors as a study in Indonesia found that risk perception had a positive influence on preventive behavior [15]. We further analyzed the factor influencing practices. Females tended to have better practices than males; this was consistent with an observation from non-travelers KAP studies [6, 10, 16–18]. As expected, the pretravel advice was found to be associated with a higher practice score toward COVID-19 among travelers; this emphasized the impact of pretravel counseling and advice. Surprisingly, while the education level was significantly associated with the knowledge score, the practice score were similar among those with a low versus high education level. This finding was dissimilar to a Chinese study, which found that the education level was correlated with a good practice [12]. There was also no correlation between the knowledge score versus the practice score, or between the attitude score versus the practice score in our study. Of note, a relatively low percentage of participants reported regular avoidance of public places/use of public transportation, cleaning of high touch surfaces every day, and checking body temperature with a thermometer if feeling unwell, all of which highlighted the need to emphasize on these preventive practices.

### Limitations

The limitations of our study included: i) limited access to travelers due to languages in the questionnaire (English/Thai); ii) a small proportion of non-Thai travelers due to unexpected travel restrictions/lock down during the observation and iii) several potential biases (from the nature of designing a study using an online questionnaire) including voluntary and convenient biases. These may affect the generalization of study results. Furthermore, the study was designed at the beginning of the outbreak, and some important information such as COVID-19 vaccination was not assessed.

## Conclusion

The study was conducted to explore the KAP regarding COVID-19 of travelers, who play a major role in the transmission of COVID-19 and in controlling the pandemic especially as international travel is resuming. This study showed that the majority of travelers had sufficient knowledge, a neutral attitude, and an acceptable practicing preventive score toward COVID-19. Social media was the major source of information, suggesting its’ significant implication in knowledge, attitude and practice promotion. Furthermore, knowledge about disease transmission should be improved. The factors influencing good practices included pretravel advice, sex, age and the point in the timeline of the outbreak. The lower knowledge and practice score in travelers participating after the end of outbreak was declared, when compared to during lockdown, suggests the importance of intensifying education and strict preventive practices over time. Pretravel counselling and advice should be encouraged in order to improve knowledge, raise awareness and emphasize good preventive measures toward COVID-19 among international travelers for improved pandemic control.

## Supplementary Information

**Additional file 1.** Site of data collection. A total of 399 international travelers and expatriates were included in the study. One hundred and forty-nine participants (37.3%) were enrolled from the Hospital for Tropical Diseases while 250 participants (62.7%) were enrolled from non-hospital areas. Among participants from outside the hospital, 177/250 participants were enrolled from state quarantine or UN soldier line groups (70.8%), the others (29.2%) were enrolled from expats groups, or Thai Travel Clinic Facebook page.

**Additional file 2.** Differences in mean knowledge score between demographic groups. Table shows differences in mean knowledge score between demographic groups.

## Data Availability

The datasets supporting the conclusions of this article are included within the article and its additional files. The raw data and the questionnaire used in this study are available at the Faculty of Tropical Medicine, Mahidol University. The corresponding author can be contacted via email: viravarn.luv@mahidol.ac.th
