# Supplementary figures and images for: Knowledge, attitude, and practices toward COVID-19 among the international travelers in Thailand
Source: Trop Dis Travel Med Vaccines. 2021 Nov 15;7:29. doi: 10.1186/s40794-021-00155-1 (PMC8590880; doi:10.1186/s40794-021-00155-1)

N = 399

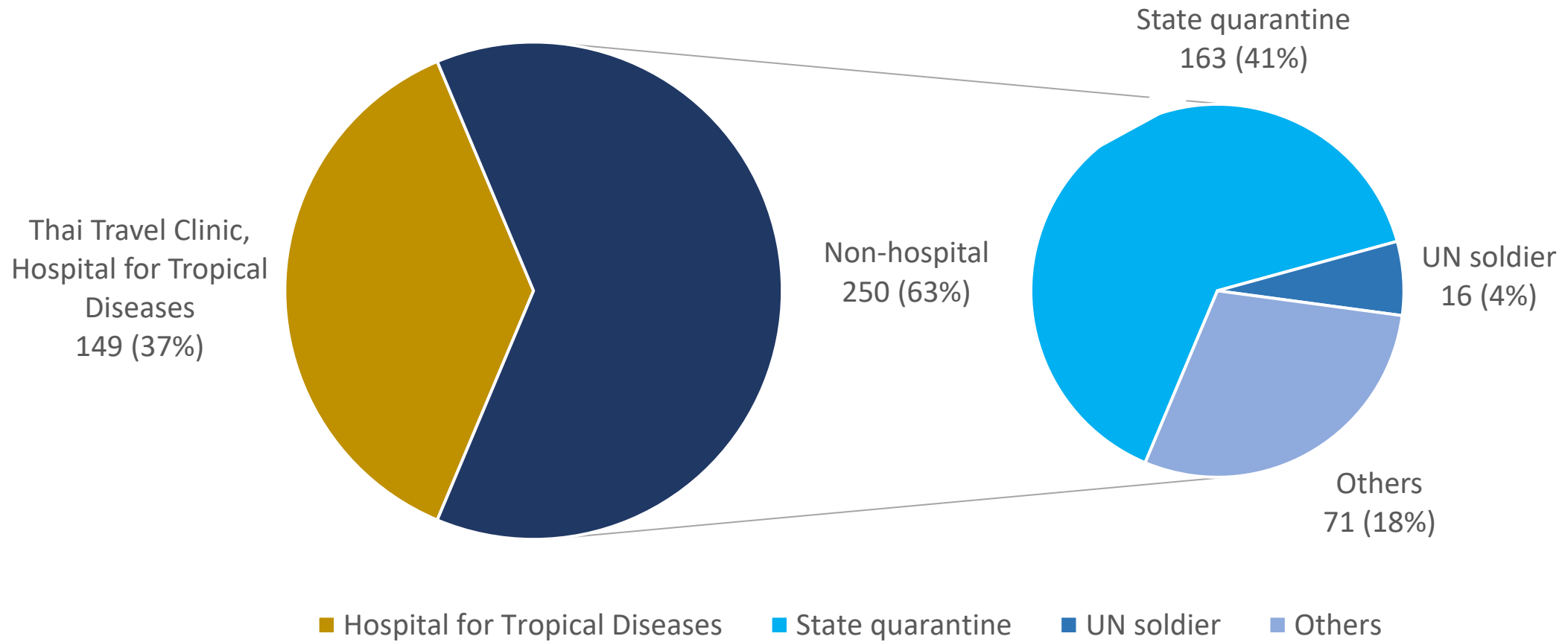

Supplement: Supplementary file 1 — Additional file 1. Site of data collection. A total of 399 international travelers and expatriates were included in the study. One hundred and forty-nine participants (37.3%) were enrolled from the Hospital for Tropical Diseases while 250 participants (62.7%) were enrolled from non-hospital areas. Among participants from outside the hospital, 177/250 participants were enrolled from state quarantine or UN soldier line groups (70.8%), the others (29.2%) were enrolled from expats groups, or Thai Travel Clinic Facebook page. [file 40794_2021_155_MOESM1_ESM.pdf]
