# Supplementary material for: Knowledge, attitude, and practices toward COVID-19 among the international travelers in Thailand
Source: Trop Dis Travel Med Vaccines. 2021 Nov 15;7:29. doi: 10.1186/s40794-021-00155-1 (PMC8590880; doi:10.1186/s40794-021-00155-1)
Supplement: Supplementary file 2 — Additional file 2. Differences in mean knowledge score between demographic groups. Table shows differences in mean knowledge score between demographic groups. [file 40794_2021_155_MOESM2_ESM.docx]

Table S1: Differences in mean knowledge scores between demographic groups

|  | N (%) | Mean ± SD | P-value |
| --- | --- | --- | --- |
| Date of data collection |  |  |  |
| - May - Jun | 224 (56.1) | 8.79 ± 1.57 | 0.042* |
| - Jul - Oct | 175 (43.9) | 8.46 ± 1.58 |  |
| - Mean difference (95% CI) |  | 0.32  (0.01, 0.64) |  |
| Site of data collection |  |  |  |
| - Hospital for Tropical Diseases | 149 (37.3) | 8.39 ± 1.60 | < 0.001* |
| - Non-hospital areas | 250 (62.7) | 8.79 ± 1.55 |  |
| - Mean difference (95% CI) |  | - 0.40  (-0.72,-0.08) |  |
| Age |  |  |  |
| - 18 – 29 | 117 (29.3) | 8.70 ± 1.76 | 0.919† |
| - 30 – 39 | 169 (42.4) | 8.65 ± 1.52 |  |
| - 40 – 49 | 76 (19) | 8.54 ± 1.56 |  |
| - 50 or above | 37 (9.3) | 8.67 ± 1.25 |  |
| Gender |  |  |  |
| - Male | 186 (46.6) | 8.69 ± 1.46 | 0.559* |
| - Female | 213 (53.4) | 8.60 ±1.67 |  |
| - Mean difference (95% CI) |  | 0.093  (-0.22, 0.40) |  |
| Traveler |  |  |  |
| - Thai | 331 (77.9) | 8.73 ± 1.55 | 0.036† |
| - Foreign | 29 (7.3) | 7.97 ± 1.70 |  |
| - Expatriate | 59 (14.8) | 8.53 ± 1.61 |  |
| Occupation |  |  |  |
| - Healthcare | 31 (7.8) | 8.48 ± 1.75 | 0.202† |
| - Non-healthcare | 256 (64.2) | 8.28 ± 1.65 |  |
| - Unemployed | 112 (28.1) | 7.99 ± 1.70 |  |
| Purpose of travel |  |  |  |
| - Leisure | 81 (20.3) | 8.12 ±1.68 | 0.002† |
| - Business/work | 175 (43.9) | 8.75 ± 1.40 |  |
| - Visiting friends or relatives | 46 (11.5) | 8.39 ± 1.91 |  |
| - Study | 60 (15) | 8.88 ± 1.56 |  |
| - Other | 37 (9.3) | 9.22 ± 1.44 |  |
| Type of travel |  |  |  |
| - Solo | 231 (57.9) | 8.53 ± 1.58 | 0.168† |
| - Tour group | 11 (2.8) | 8.73 ± 1.27 |  |
| - Travel with friends | 76 (19) | 8.63 ± 1.81 |  |
| - Business travel | 36 (9.0) | 8.81 ± 1.17 |  |
| - Travel with family | 25 (6.3) | 8.80 ± 1.63 |  |
| - Other | 20 (5.0) | 9.50 ± 1.05 |  |
| Education |  |  |  |
| - Secondary or lower | 111 (27.9) | 8.02 ± 1.67 | < 0.001* |
| - Bachelor’s or higher | 288 (72.1) | 8.89 ± 1.47 |  |
| - Mean difference |  | - 0.867  (- 1.20, -0.53) |  |
| Did you seek pretravel advice? |  |  |  |
| - Yes | 361 (90.5) | 8.63 ± 1.58 | 0.626* |
| - No | 38 (9.5) | 8.76 ± 1.55 |  |
| - Mean difference (95% CI) |  | - 0.132  (-0.66, 0.40) |  |
| Did you seek pretravel advice at hospital/clinic? |  |  |  |
| - Yes | 217 (54.4%) | 8.50 ± 1.52 | 0.043* |
| - No | 182 (45.6%) | 8.82 ± 1.63 |  |
| - Mean difference (95% CI) |  | - 0.32  (-0.63, 0.01) |  |
| Influenza vaccination in past 1 year |  |  |  |
| - Yes | 172 (43.1%) | 8.95 ± 1.45 | < 0.001* |
| - No | 227 (56.9%) | 8.41 ± 1.64 |  |
| - Mean difference (95% CI) |  | 0.53  (0.22, 0.84) |  |
| Previous COVID-19 testing |  |  |  |
| - Yes | 162 (40.6%) | 8.84 ± 1.41 | 0.035* |
| - No | 237 (59.4%) | 8.51 ± 1.67 |  |
| - Mean difference (95% CI) |  | 0.33  (0.02, 0.64) |  |

* Independent samples t-test, † One-way ANOVA
